# Effect of nonobstructive coronary stenosis on coronary microvascular dysfunction and long‐term outcomes in patients with INOCA

**DOI:** 10.1002/clc.23962

**Published:** 2022-12-25

**Authors:** Ayman A. Mohammed, Hengbin Zhang, Fuad A. Abdu, Lu Liu, Shekhar Singh, Xian Lv, Tingting Shi, Redhwan M. Mareai, Abdul‐Quddus Mohammed, Guoqing Yin, Wen Zhang, Yawei Xu, Wenliang Che

**Affiliations:** ^1^ Department of Cardiology, Shanghai Tenth People's Hospital Tongji University School of Medicine Shanghai China; ^2^ Department of Internal Medicine, Faculty of Medicine and Health Science Taiz University Taiz Yemen; ^3^ Department of Cardiology Shanghai Tenth People's Hospital Chongming branch Shanghai China

**Keywords:** CMD, INOCA, nonobstructive coronary stenosis, prognosis

## Abstract

**Background:**

Ischemic pain with no‐obstructive coronary artery (INOCA) is clinically significant and defined by nonobstructive coronary stenosis <50%. Coronary microvascular dysfunction (CMD) is a relevant cause associated with adverse outcomes.

**Objectives:**

Investigated the effect of no‐stenosis (0% stenosis) and non‐obstructive (0% < stenosis < 50%) on the prognostic impact of CMD in INOCA.

**Method:**

A retrospective study assessed the coronary microvascular function in 151 INOCA patients who underwent invasive angiography by the coronary angiography‐derived index of microcirculation‐resistance (caIMR). CZT‐SPECT was performed to evaluate myocardial perfusion imaging (MPI) abnormalities. Chi‐square test/Fisher exact test, Student *t*‐test, Kaplan–Meier curve, and Uni‐multivariable Cox proportional models were used for analysis. Clinical outcomes were major adverse cardiovascular events (MACE) during a median follow‐up of 35 months.

**Result:**

No‐stenosis was present in 71 (47%) INOCA patients, and 80 (53%) were with nonobstructive. CMD (caIMR ≥ 25) was more prevalent in patients with no‐stenosis than nonobstructive (76.1% vs. 48.8%, *p* = .001), along with abnormal MPI (39.4% vs. 22.5%, *p* = .024). The MACE rates were not different between no‐stenosis and nonobstructive stenosis. CMD showed an increased risk of MACE for all INOCA. No‐stenosis with CMD had the worst prognosis. Cox regression analysis identified CMD and abnormal MPI as predictors of MACE in all INOCA and patients with no‐stenosis. However, no‐stenosis and nonobstructive stenosis were not predictors of MACE in INOCA.

**Conclusion:**

CMD was more frequently present in INOCA with no‐stenosis. However, there was no difference in long‐term clinical outcomes between no‐stenosis and nonobstructive stenosis. CMD could independently predict poor outcomes in INOCA, particularly in patients with no‐stenosis.

## 1 INTRODUCTION

Angina pectoris affects more than 100 million people worldwide and is the most frequent symptom of ischemic heart disease.^1^ Among anginal patients with a demonstration of myocardial ischemia undergoing coronary angiography (CAG), up to 70% do not have an obstructive‐coronary disease,^2^, ^3^, ^4^ thus remaining a common challenge for physicians and patients. This condition is mainly misdiagnosed as noncardiac chest pain, so no customized treatment is prescribed for these patients. Indeed, it may cause the discontinuation of ischemic treatment inappropriately. As a result, recurrent angina occurs in these patients causing impaired life quality, recurrent hospitalizations, and short/long‐term adverse outcomes.^5^, ^6^, ^7^ The previously published studies are diverse in their definition of the absence of coronary artery disease (CAD), obstructive coronary stenosis, and prognosis.^8^ The angiographic definition of nonobstructive coronary arteries in ischemia with no obstructive coronary arteries (INOCA) utilizes the traditional cut‐off of <50% stenosis.^9^ The previous studies that investigated the prognosis and the survival rate of patients with nonobstructive coronary stenosis vary in their stratification of the coronary lesions, including totally normal (0% stenosis), normal/near normal (<20%/<30% stenosis) and nonobstructive (1/20/30%–49% stenosis) and are diverse in their results.^5^, ^8^, ^10^, ^11^, ^12^, ^13^, ^14^ Moreover, a significant proportion (more than 50%) of symptomatic INOCA patients without coronary stenotic lesions have coronary microvascular dysfunction (CMD) that contributes to myocardial ischemia.^15^, ^16^ And the presence of CMD indicates an increased risk of a worse prognosis.^17^, ^18^, ^19^ However, it is yet unclear whether non‐obstructive coronary stenosis affects the prognostic significance of CMD in INOCA.

The coronary angiogram‐derived index of microcirculation resistance (caIMR) is recently introduced as a novel noninvasive index that offers offline coronary microcirculatory evaluation without hyperemic agents. It has been shown to have a predictive value for adverse outcomes in coronary heart diseases and INOCA.^20^, ^21^, ^22^, ^23^ CZT‐SPECT myocardial perfusion imaging (MPI) is a noninvasive technique with high diagnostic accuracy of ischemia and prognostic value in cardiovascular events.^22^, ^24^, ^25^, ^26^, ^27^ Abnormal MPI findings are expected in INOCA patients with a higher risk of adverse events in those patients.^22^, ^26^, ^27^

Thus, the objective of the current study was to investigate CMD by caIMR between no‐stenosis and nonobstructive coronary stenosis in INOCA and evaluate the effect of coronary stenosis on the prognostic impact of CMD in INOCA.

## 2 METHODS

This retrospective, observational study followed the Declaration of Helsinki. The Shanghai 10th People's Hospital of Tongji University's ethical review board and committee (Shanghai, Republic of China) approved the study design. All enrolled patients signed informed consent.

### 2.1 Patient population

The inclusion criteria were (1) age ≥18 years; (2) from February 2017 to June 2019, consecutive patients with chest pain suspected to be myocardial ischemia who underwent invasive CAG and stress‐rest MPI by CZT‐SPECT within 90 days; (3) all native epicardial coronary vessels and major branches (>2.0 mm) stenosis <50%; (4) adequate quality of CAG images for caIMR measurement. Myocardial ischemia is suspected based on the clinical likelihood of ischemia (typical angina), a clinical assessment that suggests high coronary event risk, and ECG or echocardiographic findings.^2^ The exclusion criteria were (1) prior CAD defined as previous percutaneous (PCI)/surgical (CBAG) coronary revascularization or myocardial infarction (MI); (2) acute heart failure, systolic left ventricular ejection fraction <40% or Killip class IV; (3) uncontrolled hypertension; (3) unstable hemodynamically; (4) severe valvular heart disease; (5) severe renal or hepatic impairments; (6) malignancy. Baseline clinical data, risk factors, and medication intake were collected. Typical anginal chest pain was identified according to previous anginal pectoris criteria.^28^ Diabetes was diagnosed when patients were on antidiabetic therapy, fasting blood sugar ≥7.0 mmol/L or HbA1c ≥6.5%, prediabetes when fasting blood sugar (5.6–6.9 mmol/L) or HbA1c (5.7%–6.4%).

### 2.2 CAG and caIMR measurement

CAG was performed with standard techniques. Coronary luminal stenosis was determined visually by interventionists at times of CAG and later confirmed by another interventionist. According to CAG analysis, the subjects were divided into (1) a no‐stenosis group when no luminal stenosis or luminal irregularities can be seen (0% stenosis) and (2) a nonobstructive stenosis group (0% < stenosis < 50%) in the major coronary arteries or any branch.

The caIMR calculation and analysis were carried out by cardiologists who are well‐familiar and certified in using FlashAngio system software (Rainmed Ltd.). The methods of caIMR have been delineated in detail before.^20^, ^21^, ^22^ The calculation of caIMR required standard CAG images as described by Ai et al.^20^ Angiographic images with poor contrast opacification, a severe vascular overlap, disfigurement of the examined vessel, or the silhouette detection needed by the software were excluded. The final results of caIMR were assessed by two cardiologists blinded to the participant's clinical, follow‐up, and MPI information. Any conflicts were determined by consensus. caIMR was measured in at least one epicardial coronary vessel, and the vessel with the highest caIMR value among all coronary arteries was decided. Coronary microvascular dysfunction (CMD) was considered when caIMR≥25, following the current consensus of IMR.^29^, ^30^

### 2.3 CZT‐SPECT imaging protocol and analysis

A CZT‐SPECT cardiac scanner with a camera containing nine rotating pixelated locater columns of cadmium‐zinc‐telluride crystals was used to assess MPI (Spectrum Dynamics; Biosensors). For all patients, a 1‐day rest/stress protocol was employed using ^99m^Tc‐sestamibi (^99m^Tc‐MIBI). If there was no contraindication, stress‐phase testing was carried out pharmacologically by administering adenosine triphosphate (140μg/kg/min, for 6 min). The protocol applied to patients in the current study was described in detail in our group's previous study.^22^

Interpretation of all CZT‐SPECT images was achieved independently by two experienced nuclear readers of heart imaging blinded to the patient's features and outcomes data. The stress and rest phase images were interpreted visually utilizing the 17‐segments sample of the left ventricle and a five‐point measure (0 = normal, 1 = equivocal, 2 = moderate, 3 = severe decrease of radioisotope uptake, 4 = no detectable radioactive tracer in a segment).^31^ Afterward, perfusion scores were measured to show the extent and severity of abnormal myocardial perfusion. The summed stress score (SSS) is the total of all defects on the stress phase images, the summed rest score (SRS) is the total of all defects on the rest phase images, and the summed difference score (SDS) is the dissimilarity between every separate rest and stress score; these differences are then added for every segment. The stress total perfusion defect (TPD), another parameter of myocardial ischemia, was also calculated. Myocardial ischemia was considered positive in the CZT‐SPECT scan when in each coronary territory, the SSS was ≥4 and SDS was ≥2.^32^, ^33^

### 2.4 Follow‐up

The duration of follow‐up was decided from the time of the CAG to the time of the last examination. All participants were followed up by a physician's office visit or phone communication with the patients themselves or relatives and checked electronic hospital records to assess their clinical conditions. The follow up carried out by well‐trained physicians blinded to data every 6 months.

The primary endpoint of the study was composite major cardiovascular adverse events (MACE), including (1) mortality caused by clear cardiovascular events; (2) nonlethal MI; (3) unprogrammed coronary revascularization; (4) heart failure, (5) stroke, and (6) anginal‐associated rehospitalization. Cardiovascular mortality was defined as death related to myocardial infarction, fatal cardiac arrhythmia, unresponsive heart failure, cardiogenic shock, or sudden unexplained death. MI was identified as the presence of at least two of the following criteria (1) ischemic symptoms, (2) elevated cardiac enzymes, and (3) ischemic ECG changes. After the exclusion of planned procedures on the fundamental CAG's result, any coronary revascularization (with ≥50% stenosis) at the segment due to clinical deterioration was considered unprogrammed coronary revascularization. Heart failure was diagnosed based on recommended guidelines.^34^ Stroke was considered when documented thromboembolic ischemic or hemorrhagic cerebral events. Angina rehospitalization was defined by dynamic ECG alteration such as ST‐depression or T‐wave inversion with typical or equivalent chest pain without elevated cardiac enzymes and characterized by effortless symptoms, new‐onset angina (<2 months), or increase in duration/severity of previous stable angina symptoms. The first event in every patient was the MACE and was considered for the survival analysis.

### 2.5 Statistical analysis

Numerical and categorical variables were presented as mean ± SD and counted with percentages. Chi‐square test/Fisher exact test and independent Student *t*‐test were used for comparison. Kaplan–Meier curve was used to estimate the free survival of MACE, and the long‐rank test was used to detect the differences between survival curves. Uni‐multivariable Cox proportional hazard risk models were constructed to determine the risk factors of MACE. The covariates of the univariate model were age, gender, smoking, diabetes, prediabetes, hypertension, body mass index (BMI), hyperlipidemia, left ventricular systolic ejection fraction (LVEF), CMD, and abnormal MPI. The variables with *p* < .10 were adapted in the stepwise multivarieties model. The risk hazard ratio (HR) with a 95% confidence interval (CI) of the variables was calculated. Martingale residuals were used to confirm the proportional hazards assumption. The covariable numbers in the model were adjusted per the MACE number. A significant difference was considered when two‐sided *p* < .05. All statistical tests were accomplished by SPSS (version 20) and GraphPad software.

## 3 RESULTS

From February 2017 to June 2019, of the 268 enrolled patients with INOCA, 172 were prospectively enrolled in the caIMR measurements. Finally, 151 patients who met the inclusion criteria and had readable data of caIMR and CZT‐SPECT were enrolled in the current study (Supporting Information: Figure S1).

### 3.1 Baseline characteristics of INOCA patients

Of the 151 INOCA subjects (60.6 ± 9.5 years, 58.9% females), 71 (47.1%) with no‐stenosis and 80 (52.9%) with nonobstructive stenosis were enrolled in the present study. Among all INOCA, 22 (14.6%) and 70 (46.4%) were diabetics and pre‐diabetes, respectively. caIMR measurements were carried out in 337 coronary arteries: in the left anterior descending artery was 131 (38.9%), in the left circumflex artery was 98 (29.1%), and in the right coronary artery was 108 (32.0%). Patients with nonobstructive stenosis were older, had more diabetic frequency, and had less female frequency than no‐stenosis patients. Aspirin, antidiabetic medications, and statin treatment were more frequent in nonobstructive stenosis patients than in no‐stenosis patients. There was no difference between the two groups in other clinical characteristics (Table 1).

**Table 1:** Baseline characteristics of INOCA patients with no‐stenosis (0% stenosis) and nonobstructive stenosis (0% < stenosis < 50%).

Variables	No‐stenosis (*n* = 71)	Nonobstructive stenosis (*n* = 80)	*p* Value
Age (years)	58.4 ± 9.5	62.6 ± 9.1	0.006
Female, *n* (%)	53 (74.6)	36 (45.0)	<0.001
Risk factors			
BMI (kg/m^2^)	25.4 ± 3.5	24.6 ± 3.2	0.174
Smoking history, *n* (%)	7 (9.9)	16 (20.0)	0.083
Diabetes, *n* (%)	5 (7.0)	17 (21.3)	0.014
Prediabetes, *n* (%)	36 (50.7)	34 (42.5)	0.313
Hypertension, *n* (%)	21 (43.7)	45 (56.3)	0.123
Hyperlipidaemia, *n* (%)	6 (8.5)	10 (12.5)	0.420
LVEF (%)	63.2 ± 4.8	62.9 ± 3.9	0.662
Biochemical parameters			
HDL‐C (mmol/L)	1.1 ± 0.3	1.1 ± 0.2	0.235
LDL‐C (mmol/L)	2.4 ± 0.9	2.3 ± 0.9	0.318
TC (mmol/L)	4.2 ± 1.0	4.10 ± 1.1	0.420
TG (mmol/L)	1.7 ± 1.0	1.9 ± 1.6	0.462
Glucose (mmol/L)	5.2 ± 0.9	5.7 ± 1.4	0.020
HbA1c (%)	5.9 ± 0.5	6.2 ± 1.2	0.080
CRP (mg/L)	3.4 ± 5.0	3.5 ± 5.4	0.972
eGFR (ml/min/1.73m^2^)	91.1 ± 15.2	88.0 ± 15.0	0.220
NT‐proBNP (pg/ml)	174.6 ± 527.1	113.3 ± 165.2	0.334
Medication			
Aspirin, *n* (%)	33 (46.5)	50 (62.5)	0.048
Clopidogrel, *n* (%)	19 (26.8)	23 (28.8)	0.785
Beta‐blocker, *n* (%)	26 (36.6)	32 (40.0)	0.670
ACEI or ARB, *n* (%)	20 (28.2)	30 (37.5)	0.224
CCB, *n* (%)	18 (25.4)	26 (32.5)	0.335
Statin, *n* (%)	47 (66.2)	70 (87.5)	0.002
Antidiabetic medications	4 (5.6)	13 (16.3)	0.043
Physiological parameters			
caIMR	36.2 ± 14.2	29.6 ± 10.6	0.001
CMD (caIMR≥25), *n* (%)	54 (76.1)	39 (48.8)	0.001
Abnormal MPI, *n* (%)	28 (39.4)	18 (22.5)	0.024
Normal MPI, *n* (%)	43 (60.6)	62 (77.5)	0.024
Coronary affected vessels			
1‐VD, *n* (%)		44 (55.0)	
2‐VD		22 (27.5)	
3‐VD, *n* (%)		14 (17.5)	

*Note*: *p*‐value comparison between the no‐stenosis and the nonobstructive stenosis.

Abbreviations: ACEI or ARB, angiotensin‐converting enzyme inhibitor or angiotensin II receptor blocker; BMI, body mass index; caIMR, coronary angiography‑derived index of microcirculatory resistance; CCB, calcium channel blockers; CRP, C‐reactive protein; eGFR, estimated glomerular filtration rate; HDL‐C, high‐density lipoprotein; LDL‐C, low‐density lipoprotein; LVEF, left ventricular ejection fraction; MPI, myocardial perfusion imaging; NT‐proBNP, N‐terminal pro‐brain natriuretic peptide; TC, total cholesterol; TG, triglyceride; VD, vessels disease.

### 3.2 caIMR and CZT‐SPECT findings

The mean caIMR was 32.7 ± 12.8 in all INOCA patients. The caIMR was higher in no‐stenosis patients than in nonobstructive stenosis patients (36.2 ± 14.2 vs. 29.6 ± 10.6, *p* = .001). Rates of CMD were higher in no‐stenosis patients (76.1% vs. 48.8%, *p* = .001) than in nonobstructive stenosis patients. Concerning CZT‐SPECT, 46 (30.5%) patients showed abnormal MPI in all INOCA patients. The prevalence of abnormal MPI was higher in no‐stenosis (39.4% vs. 22.5%, *p* = .024) than in nonobstructive stenosis groups, Table 1. Others CZT‐SPECT findings were summarized and compared between no‐stenosis and nonobstructive stenosis groups in Supporting Information: Table S1.

### 3.3 Clinical outcomes and predictors

During the median follow‐up of 35 months (Table 2), there was 46 (30.5%) MACE in overall INOCA patients, including 2 cardiovascular mortalities, 2 unprogrammed coronary revascularizations, 9 heart failure, 12 strokes, and 21 anginal‐associated rehospitalizations. The occurrence of MACE in patients with no‐stenosis and nonobstructive stenosis was not significantly different (36.6% vs. 25%, *p* = .122). Additionally, in all INOCA patients, the MACE rate was higher in patients with CMD (40% vs. 13.8%, *p* < .001) than without CMD. Similarly, in the no‐stenosis group, the MACE rate was higher in patients with CMD (46.3% vs. 5.9%, *p* = .003) than without CMD. However, in the nonobstructive stenosis group, the MACE rate was not different between patients with CMD (33.3% vs. 17.1%, *p* = .093) and without CMD.

**Table 2:** Follow‐up data of INOCA patients with no‐stenosis (0% stenosis), nonobstructive stenosis (0% < stenosis < 50%), no‐CMD, and CMD.

	No‐stenosis (*n* = 71)	Nonobstructive stenosis (*n* = 80)	*p* Value
MACE, *n* (%)	26 (36.6)	20 (25.0)	0.122
CMD, *n* (%)	25 (46.3)	13 (33.3)	
No‐CMD, *n* (%)	1 (5.9)	7 (17.1)	
*p*‐value	0.003	0.093	
Cardiovascular death, *n* (%)	1 (1.4)	1 (1.3)	1.000
Nonfatal MI, *n* (%)	0 (0)	0 (0)	‐
Revascularization, *n* (%)	1 (1.4)	1 (1.3)	1.000
Stroke, *n* (%)	7 (9.9)	5 (6.3)	0.413
Heart failure, *n* (%)	5 (7.0)	4 (5.0)	0.735
Angina‐related rehospitalization, *n* (%)	12 (16.9)	9 (11.3)	0.316

Abbreviations: CMD, coronary microvascular dysfunction; INOCA, ischemia with no obstructive coronary arteries; MACE, major adverse cardiac events; MI, myocardial infarction.

**Figure 1:**
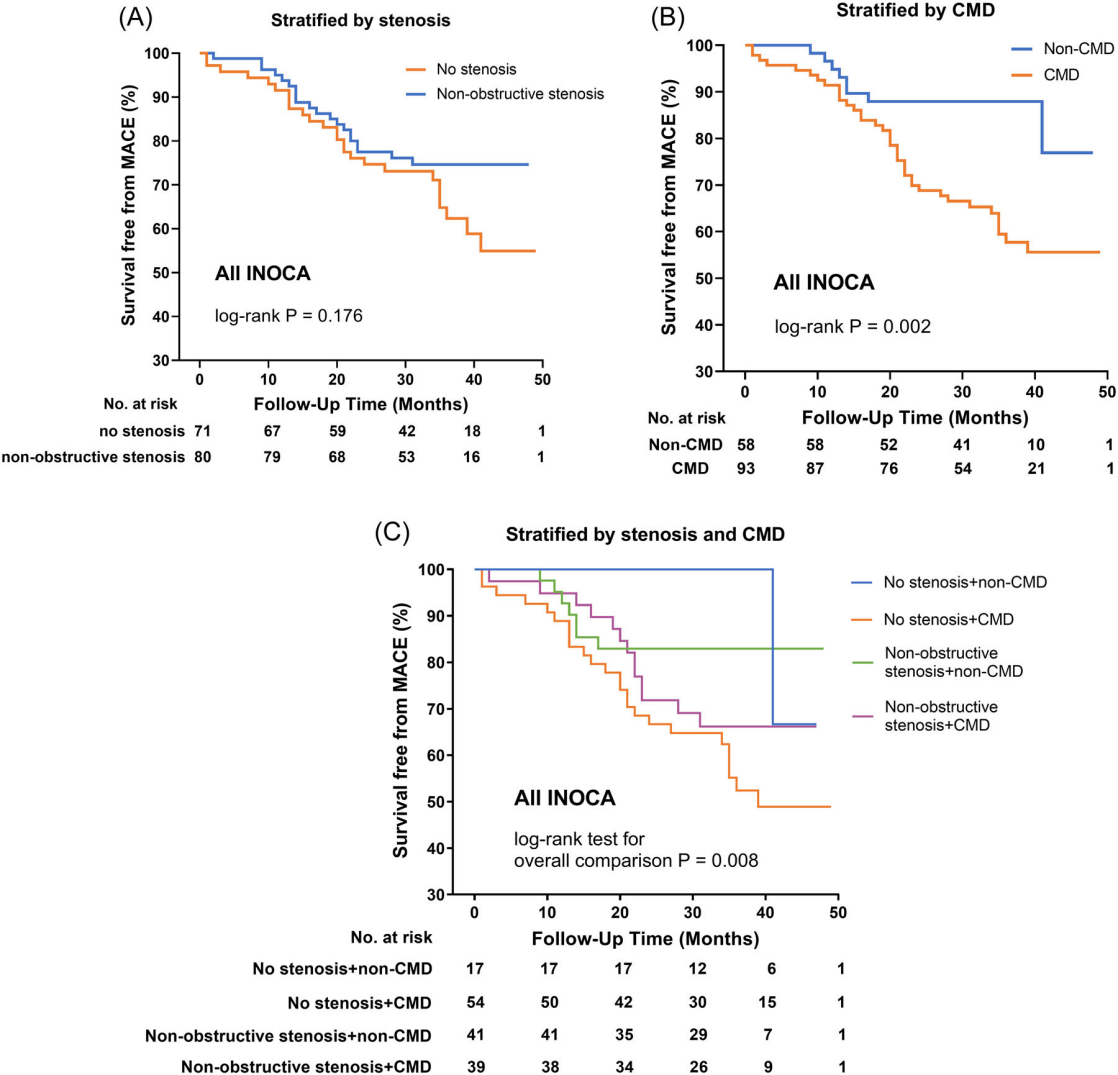
Kaplan–Meier curves for free survival from MACE in INOCA patients. (A) Kaplan–Meier curves for free survival from MACE stratified by macrovascular anatomy. (B) Kaplan–Meier curves for free survival from MACE stratified by microvascular dysfunction. (C) Kaplan–Meier curves for free survival from MACE stratified by macrovascular anatomy and microvascular dysfunction. CMD, coronary microvascular dysfunction; INOCA, ischemia with no obstructive coronary arteries; MACE, major adverse cardiac events.

**Figure 2:**
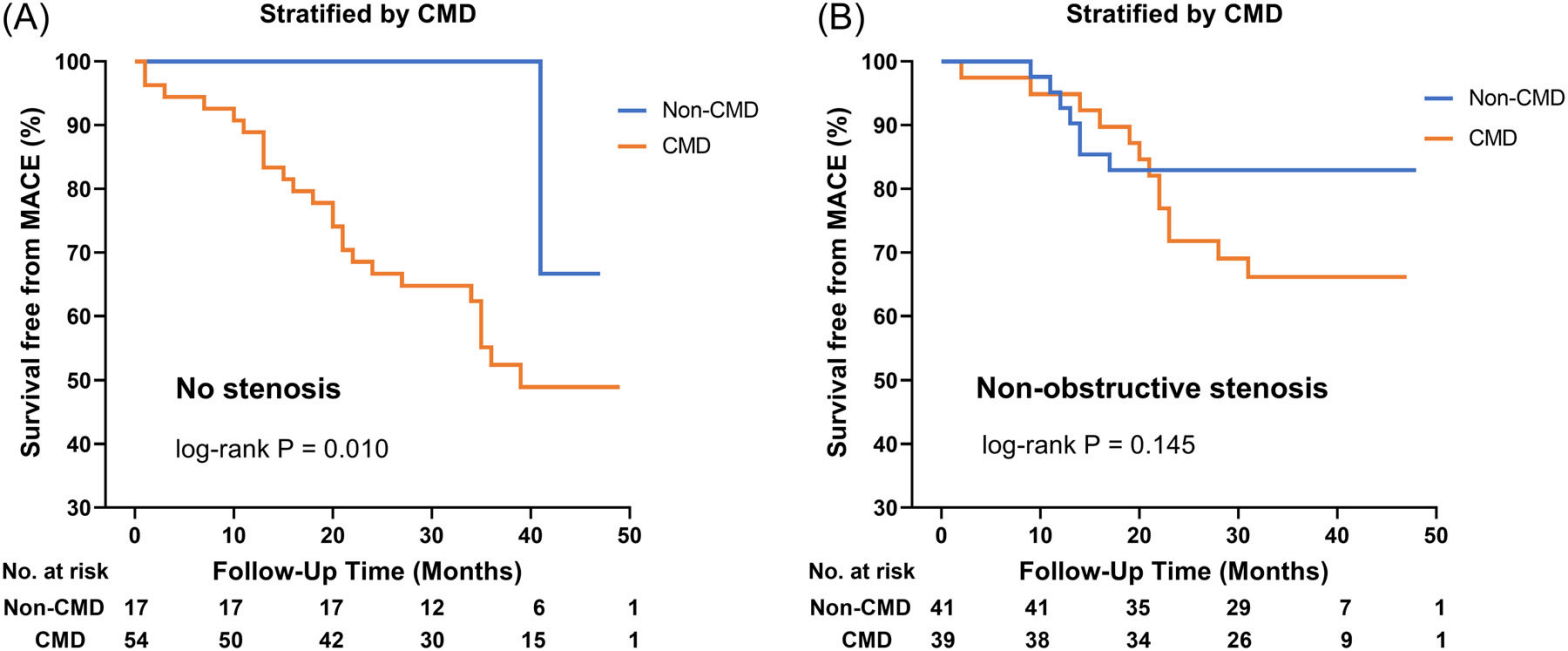
Kaplan–Meier curves for free survival from MACE among patients with no‐stenosis and nonobstructive stenosis by CMD. (A) Kaplan–Meier curve among no‐stenosis patients. (B) Kaplan–Meier curve among nonobstructive stenosis. CMD, coronary microvascular dysfunction; MACE, major adverse cardiac events.

The Kaplan–Meier analysis showed no significant increased risk of MACE in INOCA patients with no‐stenosis than with nonobstructive stenosis (log‐rank *p* = .176), Figure 1A. There was an association between CMD and free‐survival of MACE within all INOCA patients, in which patients with CMD were exposed to higher risk than non‐CMD patients (log‐rank *p* = .002), Figure 1B. When all INOCA patients were stratified by stenosis and CMD, patients with both no‐stenosis and CMD had the most increased risk than other patients (log‐rank *p* = .008), Figure 1C. Among the no‐stenosis group, CMD increased the risk of MACE more than non‐CMD (log‐rank *p* = .010), Figure 2A. However, in the nonobstructive stenosis group, CMD didn't show an increased risk of MACE than non‐CMD (log‐rank *p* = .145) (Figure 2B).

The Cox regression model in all INOCA patients showed that CMD (HR: 2.726; CI: 1.162–6.396; *p* = .021), abnormal MPI (HR: 1.979; CI: 1.030–3.801; *p* = .041) and prediabetes (HR: 2.392; CI: 1.238–4.622; *p* = .009) were independently associated with MACE in both the univariate model and multivariate model after adjusting for confounding factors. However, the no‐stenosis or nonobstructive stenosis were not associated with the prediction of MACE (*p* = .180) (Table 3). When Cox proportional hazard is constructed in the no‐stenosis group, CMD (HR: 8.535; CI: 1.089–66.894; *p* = .041), abnormal MPI (HR: 5.046; CI: 1.358–18.745; *p* = .016), prediabetes (HR: 4.341; CI: 1.013–18.607; *p* = .048) and LVEF% (HR: 0.795; CI: 0.640–0.987; *p* = .038) were independently associated with MACE, Supporting Information: Table S2A. No independent prognostic factors of MACE were found for patients with nonobstructive stenosis, Supporting Information: Table S2B.

**Table 3:** Prognostic predictors of MACE in all INOCA patients (Cox proportional hazard model)

Variables	Univariable analysis			Multivariable analysis		
	HR	95% CI	*p* value	HR	95% CI	*p* value
Age (years)	1.012	0.979–1.046	0.472			
Female	0.610	0.342–1.089	0.095	0.582	0.296–1.146	.118
BMI (kg/m^2^)	1.045	0.959–1.138	0.318			
Smoking history	0.794	0.337–1.873	0.599			
Diabetes	0.387	0.120–1.250	0.113			
Pre‐diabetes	1.965	1.086–3.555	0.025	2.392	1.238–4.622	.009
Hypertension	1.418	0.788–2.551	0.244			
Hyperlipidemia	0.811	0.291–2.263	0.690			
LVEF (%)	0.919	0.869–0.971	0.003	0.964	0.881–1.055	.429
NT‐proBNP (pg/ml)	1.001	1.000–1.001	0.006	1.001	1.000–1.001	.224
No stenosis	1.490	0.831–2.669	0.180			
CMD	3.097	1.444–6.644	0.004	2.726	1.162–6.396	.021
Abnormal MPI	2.455	1.375–4.384	0.002	1.979	1.030–3.801	.041

Abbreviations: BMI, body mass index; CI, confidence interval; CMD, coronary microvascular dysfunction; HR, hazard ratio; LVEF, left ventricular ejection fraction; MPI, myocardial perfusion imaging; NT‐proBNP, N‐terminal pro‐brain natriuretic peptide.

## 4 DISCUSSION

We investigated the prognostic influence of nonobstructive coronary stenosis on CMD in INOCA patients who underwent invasive CAG, caIMR measurement, and CZT‐SPECT MPI. The main findings are (1) the rate of CMD was higher in patients with no‐stenosis than nonobstructive stenosis, along with abnormal MPI; however, (2) there was no difference in long‐term outcomes between patients with no‐stenosis and nonobstructive stenosis, neither the no‐stenosis nor nonobstructive stenosis was a predictor for MACE; (3) there is a strong relationship between CMD and MACE events in the overall INOCA patients, and this relationship is clearly demonstrated in patients with no‐stenosis.

The nonobstructive CAD is not an uncommon finding in both acute^35^, ^36^, ^37^ and chronic^8^, ^38^ coronary syndrome in patients undergoing CAG and is associated with uncertain prognosis.^8^ Angina (symptoms and signs of cardiac ischemia) but coronary artery stenosis <50% was recognized and distinguished as INOCA.^39^ The outcome of patients with nonobstructive CAD was thought to be favorable. Until recently, many studies have shown the association between nonobstructive CAD and poor cardiovascular outcomes.^5^, ^8^, ^14^, ^38^, ^40^, ^41^

Logically one would have suspected a significantly higher percentage of adverse cardiovascular events in the nonobstructive stenosis (0% < stenosis < 50%) than in the no‐stenosis (0% stenosis) coronary arteries. Early studies showed that normal (0% stenosis) coronary lesions had a better prognosis in terms of mortality than mild (<30% stenosis), and that moderate narrowing (30%–50% stenosis) showed the worse prognosis.^10^ Another early multicentre study showed the presence of minimal coronary disease was a predictor of survival, patients with entirely normal coronary arteries (0% stenosis) had a higher 7‐year survival rate (97% vs. 92%) than those with mild stenosis (0% < stenosis < 50%), and the cardiac death rate was not significantly different between the two groups.^11^ A recent meta‐analysis identified 54 studies on angina outcomes with nonobstructive (<50% stenosis) CAD.^8^ It concluded that angina with non‐obstructive CAD had a diverse outcome, patients with less‐than‐obstructive CAD had a higher incidence of primary outcomes than totally normal coronary arteries, and patients with well‐documented myocardial ischemia (by stress echocardiography and SPECT) had the worse outcomes. In addition, some studies using coronary computed tomography angiography (CTA) to detect the degree of coronary stenosis have shown similar prognostic results; the 1%–49% luminal stenosis was associated with a higher risk of MACE than normal (0% stenosis) coronary disease.^12^, ^13^, ^38^, ^41^

In our study, there was no difference in MACE rate when patients were stratified by the degree of stenosis (0% vs. 0% < stenosis < 50%), and coronary stenosis has no predictive value for MACE, whereas CMD assessed by caIMR and abnormal MPI were independent predictors of MACE for all INOCA. Our findings are consistent with a study by Cortigiani et al.,^42^ in which CMD was assessed by echocardiography‐derived coronary flow reserve (CFR) and showed that nonobstructive (1%–40% stenosis) CAD was not a predictor of outcomes when compared with normal (0% stenosis); instead, CMD was an independent predictor. Michelle et al.^14^ also showed no difference in event rates between normal (≤20% stenosis) and nonobstructive stenosis (21%–49%), and both subgroups together had a better prognosis than obstructive stenosis (>50%) in the cardiac death and nonfatal MI. An untrue proportion of cardiovascular mortality in the no‐stenosis group due to missed lesions may explain the discrepancy between our result and the above study. Thus, the finding of visually normal‐appearing coronary arteries by angiography does not totally rule out the presence of functional or structural disorders of the coronary system, such as CMD. These findings suggest that comprehensive efforts should be made to search for underlying etiologies of ischemia by functional (noninvasive or invasive) tests.

CMD is a major factor in the pathogenesis of myocardial ischemia among patients with INOCA.^16^, ^17^ A limited number of studies investigated the relationship between CMD and stenosis in INOCA patients. CFR was higher in patients with <20% and 0% stenosis than in 20%–49% and 1%–40% stenosis, respectively,^18^, ^42^ both studies demonstrated the association of CFR with adverse outcomes among women with suspect ischemia^18^ and patients with diabetes,^42^ but these studies did not study the effect of nonobstructive stenosis on CMD. In addition, a recent study showed that angiographic coronary diameter stenosis was not associated with the presence of CMD in INOCA.^43^

Interestingly, in our study, the incidence of CMD and abnormal MPI were higher in INOCA patients, which may be implicated in the increased MACE predicted in these patients. Especially in patients with no‐stenosis and CMD with the highest risk of MACE than other patients, it could be that visually normal CAG was considered as an excellent prognosis than nonobstructive stenosis by the physician since the undertreatments were common in the no‐stenosis group.

Furthermore, in the current study, CMD and abnormal MPI were independent predictors of MACE in all INOCA patients, and this result is clearly demonstrated in patients with no‐stenosis. As previously demonstrated, CMD is strongly associated with MACE in INOCA patients. In the WISE study, the evaluation of CMD by CFR improved the prediction of MACE regardless of the presence or absence of obstructive (≥50% stenosis) CAD.^18^ In a multicenter registry of intracoronary physiologic assessment in INOCA patients, CMD was associated with an increased risk of MACE at 5 years of follow‐up.^43^

In the same manner, this study revealed that no‐stenosis with CMD had a significantly higher MACE rate than with no‐stenosis without CMD. Because the total number of CMD in the nonobstructive group was relatively small, the prognostic effect of CMD in the nonobstructive was inconclusive; thus, further studies with a larger sample are needed to verify our findings. CMD is common among diabetic^44^ and prediabetic patients.^45^ Diabetes is associated with an increased risk of adverse outcomes in patients with nonobstructive and obstructive acute MI.^46^, ^47^ In our study, the incidence of diabetes was higher in patients with nonobstructive stenosis than with no‐stenosis. However, diabetes was not a significant predictor of outcomes in univariate analysis. In contrast, prediabetes was an independent predictor of MACE in all INOCA patients and no‐stenosis INOCA patients. Therefore, future studies should explore the effect of prediabetes in INOCA patients, as antidiabetic therapy showed a reduced MACE rate among prediabetes patients with <40% coronary stenosis stable angina.^45^

Our study has some limitations: (1) It was an observational, retrospective, single‐center study with a small sample size that investigated INOCA patients who underwent CAG and DSPECT MPI. Therefore, it may not represent general clinical practice. (2) In addition, we have only used visual estimation to determine the percentage of coronary stenosis, which is often difficult to distinguish patients with 0% stenosis from those with minimal CAD on CAG alone without intravascular ultrasound or optical coherence tomography, which may cause missed lesions in the no‐stenosis group. (3) The accuracy of caIMR measurement could be influenced by the image quality, causing patient exclusion. (5) In our study, the INOCA patients did not do the intracoronary acetylcholine test; therefore, microvascular vasospasm was not assessed. (6) The duration of follow‐up was relatively short, which may have caused the under‐reporting of the MACE rate. Multicenter prospective studies with intracoronary imaging testing to evaluate the degree of stenosis and assess microvascular endothelial (microvascular vasospasm) function, with a longer follow‐up, are necessary to ascertain our findings.

## 5 CONCLUSION

CMD was more frequently present in INOCA with no‐stenosis; however, there was no difference in long‐term clinical outcomes between no‐stenosis and nonobstructive stenosis. CMD could independently predict poor outcomes in INOCA, particularly in patients with no‐stenosis. In INOCA patients, further functional investigations for CMD are justified in risk stratification.

## AUTHOR CONTRIBUTIONS

*Conception and design*: Lu Liu, Ayman A. Mohammed, Hengbin Zhang, Wenliang Che; *Administrative support*: Fuad A. Abdu, Wenliang Che; *Provision of study materials or patients*: Shekhar Singh, Xian Lv, Tingting Shi; *Collection and assembly of data*: Redhwan M. Mareai, Abdul‐Quddus Mohammed, Guoqing Yin; *Data analysis and interpretation*: Lu Liu, Ayman A. Mohammed, Hengbin Zhang; *Manuscript writing*: All authors; *Final approval of manuscript*: All authors.

## CONFLICT OF INTEREST

The authors declare no conflict of interest.

## Supporting information

Supplementary information.

Supplementary information.

## Data Availability

Data available on request to the corresponding author due to privacy/ethical restrictions.
