# Supplementary material for: Effect of nonobstructive coronary stenosis on coronary microvascular dysfunction and long‐term outcomes in patients with INOCA
Source: Clin Cardiol. 2022 Dec 25;46(2):204–13. doi: 10.1002/clc.23962 (PMC9933113; doi:10.1002/clc.23962)
Supplement: Supplementary file 1 — Supplementary information. [file CLC-46-204-s001.docx]

**Table 1.** CZT SPECT MPI data of INOCA patients with no stenosis (0% stenosis) and non-obstructive stenosis (0%< stenosis <50%).

| Variables | No stenosis (n=71) | Non-obstructive stenosis (n=80) | *P-value* |
| --- | --- | --- | --- |
| Abnormal MPI, n (%) | 28(39.4) | 18(22.5) | 0.024 |
| Normal MPI, n (%) | 43(60.6) | 62(77.5) | 0.024 |
| SSS (median ± IQR) | 2 ± 6 | 1 ± 3 | 0.006 |
| SRS (median ± IQR) | 0 ± 1 | 0 ± 0 | 0.103 |
| SDS (median ± IQR) | 2 ± 4 | 1 ± 2 | 0.008 |
| Stress TPD (%) | 3.56 ± 3.33 | 1.96 ± 2.22 | 0.001 |
| Stress perfusion defect location |  |  |  |
| Anterior, n (%) | 29(40.8) | 18(22.5) | 0.015 |
| Lateral, n (%) | 27(38.0) | 16(20.0) | 0.014 |
| Inferior or posterior, n (%) | 17(23.9) | 19(23.8) | 0.978 |
| Septum, n (%) | 17(23.9) | 19(23.8) | 0.978 |
| Apical, n (%) | 7(9.9) | 10(12.5) | 0.608 |
| Rest perfusion defect location |  |  |  |
| Anterior, n (%) | 10(14.1) | 5(6.3) | 0.108 |
| Lateral, n (%) | 5(7.0) | 4(5.7) | 1.000 |
| Inferior or posterior, n (%) | 4(5.6) | 6(7.5) | 0.750 |
| Septum, n (%) | 3(4.2) | 8(10.0) | 0.219 |
| Apical, n (%) | 3(4.2) | 1(1.3) | 0.342 |
| Coronary territory of the defects |  |  |  |
| LAD, n (%) | 42(59.2) | 39(48.8) | 0.201 |
| LCX, n (%) | 33(46.5) | 16(20.0) | 0.001 |
| RCA, n (%) | 26(36.6) | 24(30.0) | 0.388 |
| Left ventricular functional parameters | |  |  |
| Stress EDV (ml) | 69.24 ± 23.74 | 72.06 ± 23.78 | 0.467 |
| Stress ESV (ml) | 24.62 ± 16.43 | 26.25 ± 15.50 | 0.532 |
| Stress LVEF (%) | 66 ± 11 | 66 ± 9 | 0.789 |
| Stress PER(-EDV/s) | 3.46 ± 0.70 | 3.45 ± 0.70 | 0.907 |
| Stress PFR(EDV/s) | 2.56 ± 0.90 | 2.62 ± 2.50 | 0.847 |
| Rest EDV (ml) | 64.27 ± 23.76 | 67.60 ± 25.16 | 0.406 |
| Rest ESV (ml) | 21.89 ± 17.04 | 23.41 ± 16.77 | 0.581 |
| Rest LVEF (%) | 68 ± 12 | 68 ± 12 | 0.931 |
| Rest PER(-EDV/s) | 3.55 ± 0.67 | 3.63 ± 0.78 | 0.493 |
| Rest PFR(EDV/s) | 2.53 ± 0.68 | 2.48 ± 0.65 | 0.668 |
| ∆LVEF (%) | 2 ± 11 | 2 ± 9 | 0.803 |
| TID | 1.11 ± 0.13 | 1.09 ± 0.14 | 0.350 |

*MPI*, myocardial perfusion imaging; *SSS*, summed stress score; *SRS*, summed rest score; *SDS*, summed difference score; *TPD*, total perfusion defects; *EDV*, end-diastolic volume; *ESV*, end-systolic volume; *LVEF*, left ventricular ejection fraction; *PER*, peak ejection rate; *PFR*, peak filling rate; *TID*, transient ischemic dilation.

*P* value comparison between the No stenosis and the non-obstructive stenosis.

**Table 2. A.** Prognostic predictors of MACE in INOCA patients with no stenosis (Cox proportional hazard model)

| Variables | Univariable Analysis | | |  | Multivariable Analysis | | |
| --- | --- | --- | --- | --- | --- | --- | --- |
|  | HR | 95% CI | *P-value* |  | HR | 95% CI | *P-value* |
| Age (years) | 1.029 | 0.983 - 1.078 | 0.219 |  |  |  |  |
| Female | 0.529 | 0.236 - 1.187 | 0.123 |  |  |  |  |
| BMI (kg/m^2^) | 1.058 | 0.940 - 1.190 | 0.353 |  |  |  |  |
| Smoking history | 1.125 | 0.337 - 3.757 | 0.848 |  |  |  |  |
| Diabetes | 0.043 | 0.000 - 21.277 | 0.320 |  |  |  |  |
| Pre-diabetes | 2.114 | 0.940 – 4.757 | 0.070 |  | 4.341 | 1.013 – 18.607 | 0.048 |
| Hypertension | 1.743 | 0.800 - 3.798 | 0.162 |  |  |  |  |
| Hyperlipidaemia | 0.403 | 0.055 - 2.979 | 0.373 |  |  |  |  |
| LVEF (%) | 0.918 | 0.862 - 0.977 | 0.007 |  | 0.795 | 0.640 - 0.987 | 0.038 |
| NT-proBNP (pg/mL) | 1.001 | 1.000 - 1.001 | 0.025 |  | 0.999 | 0.997 - 1.001 | 0.318 |
| CMD | 8.782 | 1.189 - 64.878 | 0.033 |  | 9.007 | 1.040 - 78.037 | 0.046 |
| Abnormal MPI | 2.648 | 1.196 - 5.862 | 0.016 |  | 3.438 | 1.118 - 10.575 | 0.031 |
| **B.** Prognostic predictors of MACE in INOCA patients with non-obstructive stenosis (Cox proportional hazard model) | | | | | | | |
| Variables | Univariable Analysis | | |  | Multivariable Analysis | | |
|  | HR | 95% CI | *P-value* |  | HR | 95% CI | *P-value* |
| Age (years) | 1.004 | 0.952 - 1.058 | 0.888 |  |  |  |  |
| Female | 0.491 | 0.189 - 1.280 | 0.146 |  |  |  |  |
| BMI (kg/m^2^) | 1.008 | 0.883 - 1.150 | 0.907 |  |  |  |  |
| Smoking history | 0.658 | 0.193 - 2.246 | 0.504 |  |  |  |  |
| Diabetes | 0.637 | 0.187 - 2.176 | 0.472 |  |  |  |  |
| Pre-diabetes | 1.736 | 0.719 – 4.191 | 0.220 |  |  |  |  |
| Hypertension | 1.160 | 0.474 - 2.840 | 0.745 |  |  |  |  |
| Hyperlipidaemia | 1.281 | 0.375 - 4.374 | 0.693 |  |  |  |  |
| LVEF (%) | 0.899 | 0.800 - 1.010 | 0.072 |  |  |  |  |
| NT-proBNP (pg/mL) | 1.002 | 1.000 - 1.003 | 0.112 |  |  |  |  |
| CMD | 1.950 | 1.778 - 4.888 | 0.155 |  |  |  |  |
| Abnormal MPI | 1.953 | 0.779 - 4.897 | 0.154 |  |  |  |  |

*HR*, hazard ratio; *CI*, confidence interval; *BMI*, body mass index; *LVEF*, left ventricular ejection fraction; *NT-proBNP*, N-terminal pro-brain natriuretic peptide; *CMD*, coronary microvascular dysfunction; *MPI*, myocardial perfusion imaging.
