# Supplementary material for: Effect of nonobstructive coronary stenosis on coronary microvascular dysfunction and long‐term outcomes in patients with INOCA
Source: Clin Cardiol. 2022 Dec 25;46(2):204–13. doi: 10.1002/clc.23962 (PMC9933113; doi:10.1002/clc.23962)
Supplement: Supplementary file 2 — Supplementary information. [file CLC-46-204-s002.docx]

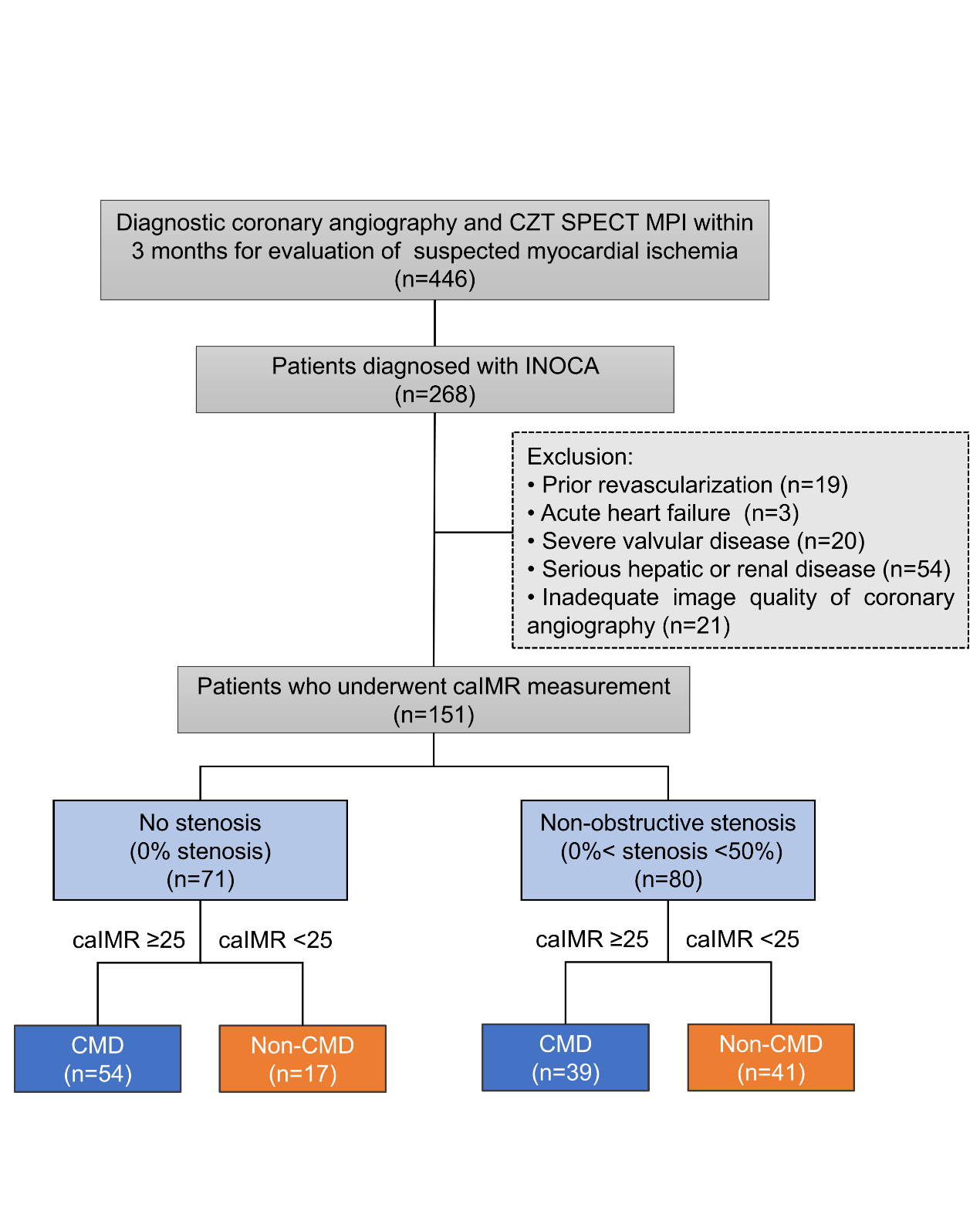


Figure 1: Study Flow chart.

Abbreviations: MPI, myocardial perfusion imaging; INOCA, ischemia with non-obstructive coronary artery disease; caIMR, coronary angiography‑derived index of microcirculatory resistance; CMD, coronary microvascular dysfunction.
